# The partial dissociation of MHC class I–bound peptides exposes their N terminus to trimming by endoplasmic reticulum aminopeptidase 1

**DOI:** 10.1074/jbc.RA117.000313

**Published:** 2018-03-29

**Authors:** Athanasios Papakyriakou, Emma Reeves, Mary Beton, Halina Mikolajek, Leon Douglas, Grace Cooper, Tim Elliott, Jörn M. Werner, Edward James

**Affiliations:** From the ‡ Centre for Biological Sciences, Faculty of Natural & Environmental Sciences, University of Southampton, Southampton SO17 1BJ, United Kingdom,; § Institute for Life Sciences, University of Southampton, Southampton SO17 1BJ, United Kingdom, and; ¶ Centre for Cancer Immunology, University of Southampton Faculty of Medicine, University Hospital Southampton, Southampton SO16 6YD, United Kingdom

**Keywords:** antigen processing, antigen presentation, major histocompatibility complex (MHC), molecular dynamics, X-ray crystallography, ERAP1, free energy calculations, H2-Kb, peptide trimming, single chain trimer

## Abstract

Endoplasmic reticulum aminopeptidase 1 (ERAP1) and ERAP2 process N-terminally extended antigenic precursors for optimal loading onto major histocompatibility complex class I (MHC I) molecules. We and others have demonstrated that ERAP1 processes peptides bound to MHC I, but the underlying mechanism is unknown. To this end, we utilized single-chain trimers (SCT) of the ovalbumin-derived epitope SIINFEKL (SL8) tethered to the H2-K^b^ MHC I determinant from mouse and introduced three substitutions, E63A, K66A, and W167A, at the A-pocket of the peptide-binding groove in the MHC I heavy chain, which interact with the N termini of peptides. These variants significantly decreased SL8-presenting SCT at the cell surface in the presence of ERAP1, but did not affect overall SCT expression, indicating that ERAP1 trims the SL8 N terminus. Comparison of the X-ray crystal structures of WT and three variant SCTs revealed only minor perturbations of the peptide-binding domain in the variants. However, molecular dynamics simulations suggested that SL8 can dissociate partially within a sub-microsecond timescale, exposing its N terminus to the solvent. We also found that the C terminus of MHC I–bound SL8 remains deeply buried in the F-pocket of MHC I. Furthermore, free-energy calculations revealed that the three SCT variants exhibit lower free-energy barriers of N terminus dissociation than the WT K^b^. Taken together, our results are consistent with a previously observed model in which the partial dissociation of bound peptides from MHC I exposes their N terminus to trimming by ERAP1, whereas their C terminus is anchored at the F-pocket.

## Introduction

Major histocompatibility complex class I (MHC I)^3^ molecules present a diverse array of antigenic peptides, the so-called immunopeptidome, to circulating cytotoxic T lymphocytes. MHC Is are heterotrimers consisting of a transmembrane heavy chain (HC), β_2_-microglobulin (β_2_m), and a peptide antigen, typically of 8–11 amino acids in length. These peptides originate from self-proteins including aberrantly expressed cancer antigens as well as foreign proteins from intracellular pathogens. The precise composition of the immunopeptidome and its ability to shape the antigen-specific T cell response to both infections and cancer are thus central to the mechanism of immunosurveillance.

The antigen processing pathway consists of a number of sequential steps and editing events to ensure complete and stable assembly of peptide–MHC I (pMHC I) complexes in the endoplasmic reticulum (ER) and their expression at the cell surface. Antigenic precursors are generated from larger protein fragments by the (immuno)proteasome and may undergo further processing by cytosolic proteases (1–3). The resulting peptides often contain families of N-terminally extended precursors which require further processing before they can bind to MHC I with high affinity (2–4). These precursors are transported into the ER lumen by the transporter associated with antigen processing (TAP) with a preference for 8- to 12-mer peptides (5, 6). Here, endoplasmic reticulum aminopeptidase 1 (ERAP1) and ERAP2 trim peptides to the optimal length for stable MHC I binding in the final editing step of the antigen processing pathway (7–9). The peptide-loading complex (PLC) in the ER orchestrates peptide transport and the assembly of newly synthesized MHC I with high-affinity peptides, a process that is tightly regulated by the peptide-editing function of tapasin (Tpn), resulting in the preferential egress to the cell surface of stable pMHC I (10). The editing functions of ERAP1/2 and Tpn are synchronized spatiotemporally, although no physical interaction between ERAP1/2 and Tpn has yet been established.

The trimming function of ERAP1 shapes the immunopeptidome; genetic knockout of ERAP1 (ERAAP in mice) reduces the level of MHC I expression at the cell surface by up to 70% in mice and 10% in humans, depending on the MHC I allele (7, 11). This loss of ERAP1 function gives rise to fewer peptides that are longer in length (12, 13). The resultant change in repertoire can initiate a strong cytotoxic T lymphocyte (CTL) response *in vivo* if ERAAP KO cells are injected into ERAAP-competent mice, indicating the ability of ERAAP/ERAP1 to control the epitope specificity of CTL responses (14). In accordance with TAP transport, ERAP1 has a substrate length preference of 10- to 16-mer peptides (15) and trims different amino acid residues from the N terminus of precursor peptides at different rates, thereby forming a distinct hierarchy of N-terminal extension preferences (16, 17). Through the combined action on candidate peptides of N-terminal trimming and MHC I binding, the dominant products are peptides of 8–10 amino acids, which is generally optimal for forming stable pMHC I complexes. Importantly, ERAP1 is also able to “overtrim” peptides, resulting in the destruction of some epitopes (11, 18, 19). This specificity in turn shapes the abundance of peptides presented, and can affect the magnitude of T cell responses to pathogens and cancer (18, 20). Thus, ERAP1 is a key regulator of peptide generation and presentation to CTL.

In humans, ERAP1 is highly polymorphic, with single nucleotide polymorphisms (SNPs), associated with a number of autoimmune diseases and cancer (21–24). SNPs in ERAP1 form discrete allotypes, which are associated with different trimming specificities broadly categorized into efficient, hypoactive, and hyperactive phenotypes. Allotype-dependent ERAP1 trimming activity affects the peptide repertoire and the level of MHC I at the cell surface, and these activities combine in heterozygotes (17, 25). The structural basis for the differences in function because of the SNPs is not clear. Some polymorphic sites may interact with parts of the active site, an obvious target for altered function, whereas others may interfere with the dynamics of the “open” and “closed” conformation (26–28). However, the majority of polymorphic residues are not located within the active site or interdomain junctions, and are harder to reconcile with function.

The precise mechanism by which ERAP1 trims N-terminal amino acids remains unclear. Although ERAP1 has not been shown to interact directly with the PLC, studies have identified the requirement of MHC I for ERAP1 peptide trimming, resulting in destruction of the epitope when the correct MHC I is absent (29). Interestingly, we and others have shown the ability of ERAP1 to process peptides bound to MHC I (17, 30). This suggests that the substrate for ERAP1 could be a pMHC I complex in which (*a*) the peptide N terminus is permanently or occasionally exposed to the ERAP1 active site as a result of N-terminal extension out of the A-pocket of the peptide binding groove (in which the N terminus of bound peptides are buried in the native pMHC I structure), or (*b*) suboptimal docking of the peptide N terminus with the binding groove. A complementary or alternative hypothesis has been suggested, supported by *in vitro* and crystallographic studies, where ERAP1 acts as the “molecular ruler” by binding both the N and C termini of a free peptide within an internal substrate-binding channel (27, 31).

To establish proof of principle that the ability of ERAP1 to trim MHC I–bound peptide depended on an exposed N terminus we investigated ERAP1 peptide trimming after mutating the MHC I A-pocket in such a way as to reduce the likelihood of its forming stable interactions with the N terminus of bound peptide and protecting it from trimming. We show that pMHC I complexes with single point mutations at key peptide-interacting residues in the A-pocket, and engineered for enhanced binding to peptide at its C terminus, result in greater susceptibility to ERAP1 overtrimming.

## Results

### Mutation of A-pocket residues enhances ERAP1-dependent trimming of bound peptide

ERAP1 has been shown to trim peptides bound to MHC I, however, the precise mechanism by which this occurs is still unknown. Nevertheless, these studies suggest that MHC I may act as a molecular template for trimming of peptides to the correct length for presentation, and may be regulated by the relative kinetics of ERAP1 trimming *versus* A-pocket binding for a peptide bound in the peptide-binding groove (17, 30). A greater understanding of the mechanism that underpins the trimming of MHC I–bound peptide may help to interpret the observed variations in activity between ERAP1 allotypes, including overtrimming, which are harder to accommodate within the ERAP1 molecular ruler hypothesis.

**Figure 1.**
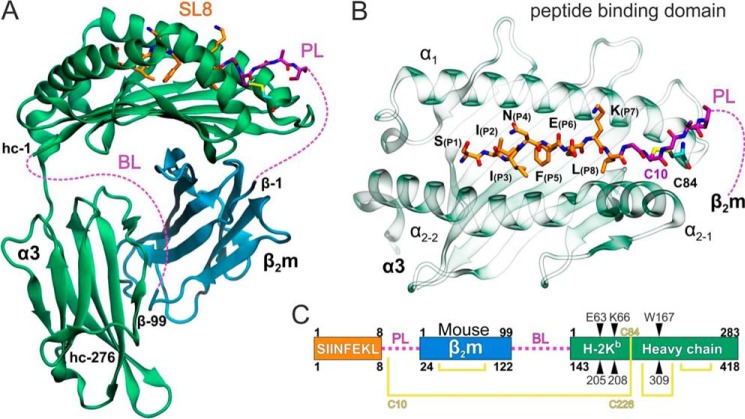
**Disulfide trap single-chain trimer structure.** *A*, ribbon representation of the WT-SCT color-coded with *green* for the HC and *blue* for the β_2_m domain. SL8 is shown with *orange* C sticks and the linker between SL8 and β_2_m (*PL*) with *purple* C atoms. PL residues that were not resolved in our X-ray structures and the linker between β_2_m and the K^b^ heavy chain (*BL*) are indicated by *purple dashed lines*. The N and C termini of HC and β_2_m are indicated as hc1–hc276 and β1–β99, respectively. *B*, close-up view of the peptide-binding domain illustrating SL8 (*orange* C, *blue* N, *red* O), the PL residues resolved (*purple* C), and the engineered residue Y84C (*cyan* C, *yellow* S) that comprises the disulfide trap with the PL residue Cys^10^ (*C10*). *C*, schematic organization of the WT-SCT showing the residue numbering of each domain (*upper*) and the corresponding numbering in the PDB files (*lower*). Disulfide bridges are shown with *yellow lines* and the three mutated positions are indicated.

Based on an examination of the native structure of the SIINFEKL (SL8)/K^b^ pMHC I complex (32), and the structure of the single-chain SL8-β_2_m-K^b^ HC trimer (33), we introduced alanine substitutions at residues 63, 66, and 167 in the MHC I HC which interact with the bound peptide in the A-pocket. Positions Glu^63^ and Lys^66^ interact directly with S(P_1_) and I(P_2_) of SL8, whereas Trp^167^ caps off the N terminus amino group of the peptide (32, 33) (Fig. S1). Alanine substitutions at the three K^b^ residues would be expected to significantly reduce the number of hydrogen bonds and van der Waals contacts between the peptide and peptide-binding groove, thus reducing the binding affinity of the N terminus of SL8 to the A-pocket of K^b^. To avoid complications arising from consequent lowering of the macroscopic off-rate, we utilized a disulfide-linked SL8/K^b^ single chain construct (WT-SCT) (Fig. 1). The construct consists of SL8 peptide linked with β_2_m and K^b^ HC in which the C terminus of SL8 is tethered to the K^b^-binding groove through a disulfide bond between Y84C of K^b^ and a second cysteine within the peptide-β_2_m linker (Fig. 1, *B* and *C*) (34).

**Figure 2.**
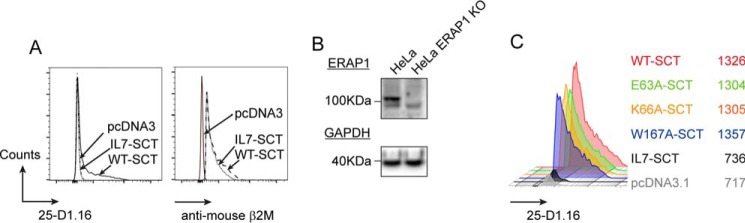
**Cell surface expression of WT and A-pocket variant SCT.** HeLa and HeLa ERAP1 KO (E1KO) cells were transfected with WT-SCT, A-pocket variants E63A-, K66A-, or W167A-SCT and cell surface expression assessed using the SIINFEKL/K^b^-specific antibody 25-D1.16 or anti-mouse β2m. *A*, 25-D1.16 and anti-mouse β2m staining of HeLa cells transfected with either WT-SCT or IINFEKL (IL7)-SCT. *B*, CRISPR/Cas9 knockout of ERAP1 in HeLa cells. *C*, 25-D1.16 staining of HeLa E1KO cells transfected with WT or A-pocket variant SCT.

We have previously demonstrated that the WT-SCT can be trimmed to IINFEKL/K^b^ (IL7-SCT) by a hyperfunctional ERAP1 variant using mass spectrometric analysis (17). Another way of detecting this trimming event is to use recognition of the pMHC I complex by the 25-D1.16 antibody, which is specific for the SL8/K^b^ complex and recognizes WT-SCT (35). We confirmed this reactivity and in addition showed that 25-D1.16 did not recognize IL7-SCT when transfected into HeLa cells despite being expressed at similar levels to the WT-SCT control, as detected by anti-mouse β_2_M antibody (Fig. 2*A*). This indicated that 25-D1.16 reactivity could be used as an indirect measure of ERAP1-dependent N-terminal trimming of MHC-bound SL8 in the WT-SCT, offering a convenient way to determine whether mutations in the A-pocket of K^b^ permitted “overtrimming” of its peptide cargo by ERAP1.

To evaluate the impact of mutating A-pocket side chains on the N-terminal trimming by ERAP1, we first generated an ERAP1 knockout in HeLa cells using CRISPR/Cas9 (HeLa E1KO). This approach resulted in cells expressing no detectable ERAP1 protein (Fig. 2*B*). Transfection of HeLa E1KO with WT-SCT or each of the three variant SCTs resulted in their expression at the cell surface at similar levels measured by 25-D1.16 (Fig. 2*C*). This confirmed that mutation of the A-pocket does not affect the ability of the variants to fold in the ER and egress to the cell surface, which is consistent with the observation that there is no interaction between 25-D1.16 and Glu^63^, Lys^66^ or Trp^167^ in the crystal structure of the SL8/K^b^/25-D1.16 complex (36).

**Figure 3.**
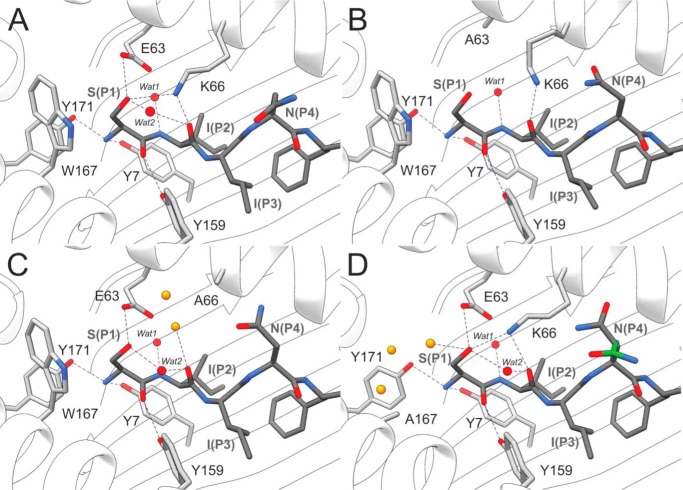
**The effect of A-pocket mutation on SCT peptide-binding groove structure.** *A–D*, close-up view of the peptide-binding domain of the WT-SCT crystal structures illustrating hydrogen-bonding interactions between S(P_1_), I(P_2_), and the mutated residues in (*A*) WT, (*B*) E63A, (*C*) K66A, and (*D*) W167A variants. Solvent molecules that mediate conserved hydrogen bonds are shown as *red spheres* (Wat1/2), and *yellow spheres* indicate waters that fill a space created by the mutated residue. The second alternative conformation of the N(P_4_) side chain resolved in the W167A-SCT (*D*) is highlighted with *green* C atoms.

To confirm that the A-pocket mutations did not disrupt the general architecture of the peptide-binding groove of K^b^, we determined the structures of WT-SCT (Fig. 1) and its three alanine-substituted variants by X-ray crystallography. The structures were resolved to a resolution range of 1.90–2.40 Å (Table 1) and showed the same topologies for β_2_m, α_3_, and peptide-binding domains. In line with the structural similarity of the backbone between WT and variants, the previously identified hydrogen bonds between (i) the terminal amine of S(P_1_) and the phenolic groups of Tyr^7^ and Tyr^171^, (ii) the backbone carbonyl of S(P_1_) and the phenolic group of Tyr^159^, and (iii) the amide of I(P_2_) with a structurally conserved water molecule (Wat1) remain preserved in all variants (Fig. 3). A hydrogen bond between the S(P_1_) hydroxyl group and the side chain carboxylate group of Glu^63^ is detected in WT-, W167A-, and K66A-SCT, but not in the E63A-SCT variant (Fig. 3*B*). Similarly, the hydrogen bond between the backbone carbonyl group of I(P_2_) and the side chain amine of Lys^66^, present in WT-, E63A-, and Trp^167^-SCT, is not present in K66A-SCT (Fig. 3*C*). The space created by the shorter Ala^66^ side chain is occupied by two water molecules, one of which is stabilized by a hydrogen bond with the carbonyl group of I(P_2_). For the W167A-SCT variant the structure and H-bonding network around S(P_1_) and I(P_2_) remain very similar to WT-SCT, whereas the space created by the missing Trp^167^ indole ring is filled by three water molecules (Fig. 3*D*). Despite the loss of hydrogen bonds, there was little variation in the peptide conformation and the interacting residues of the peptide-binding groove varied very little; the only obvious differences being in the orientation of the side chains of Lys^66^ and N(P_4_) in the E63A-SCT (Fig. 3*B*). Interestingly, the side chain of N(P_4_) adopted similar orientation in K66A-SCT, whereas two alternative rotamer states of the N(P_4_) side chain were resolved in W167A-SCT (Fig. 3, *C* and *D*). Such side chain rearrangements, although minor, are suggestive of an increased flexibility in SL8 within the peptide-binding groove.

**Table 1:** **Data collection statistics and refinement for the SL8-K^b^ single-chain trimers**

	Protein			
	Wildtype (5OQF)	E63A (5OQI)	K66A (5OQH)	W167A (5OQG)
Space group	P 21	P 21	P 21	P 21
Unit cell				
a, b, c (Å)	66.65, 90.08, 89.33	67.13, 90.19, 89.45	66.43, 89.32, 89.40	66.43, 89.64, 89.45
α, β, γ (°)	90.00, 111.23, 90.00	90.00, 111.22, 90.00	90.00, 111.24, 90.00	90.00, 111.24, 90.00
Upper resolution limit (Å)	2.27	2.40	2.05	1.90
Lower resolution limit (Å)	90.08	42.52	39.36	61.51
Multiplicity	2.8	3.8	3.8	7.5
Completeness (%)	98.6	97.9	98.2	99.8
*R*_merge_ (%)	7.7	10.3	7.4	7.0
Mean (*I*)/S.D. (*I*)	9.8 (2.0)	11.6 (2.1)	9.9 (2.4)	13.8 (2.8)
Reflections	44,965	38,114	59,972	76,876
*R*_factor_	0.199	0.198	0.204	0.188
*R*_free_	0.250	0.259	0.241	0.239
Crystallization conditions	Ammonium sulfate, PEG 8K, sodium cacodylate, pH 6.0	PEG 4K, sodium cacodylate, pH 6.5	Ammonium sulfate, PEG 8K, sodium cacodylate, pH 6.0	Ammonium sulfate, PEG 8K, bis-tris, pH 6.5
Average all-atom B-factor (Å^2^)	38.0	38.0	35.0	38.0
RMSD^Cα^ against WT (Å) for chain A/B		0.514/0.413	0.266/0.404	0.355/0.391
Peptide RMSD^Cα^ against WT (Å) for chain A/B		0.268/0.223	0.268/0.179	0.180/0.248
RMSD^Cα^ against 2QRT (Å) for chain A/B	0.401/0.449	0.522/0.445	0.375/0.295	0.414/0.388
Peptide RMSD^Cα^ against 2QRT (Å) for chain A/B	0.239/0.394	0.329/0.389	0.145/0.344	0.192/0.280

**Figure 4.**
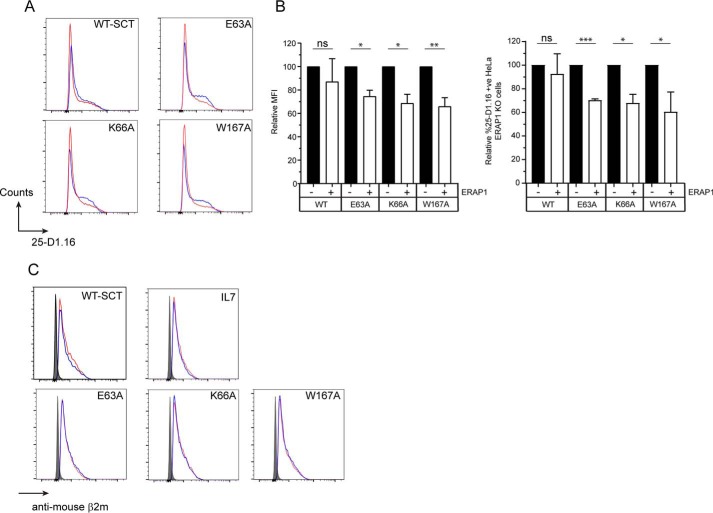
**A-pocket mutations allow trimming of SIINFEKL peptide bound to K^b^.** HeLa ERAP1 KO cells were transfected with WT-SCT, A-pocket variants E63A-, K66A- or W167A-SCT with or without the overexpression of ERAP1. Cell surface expression of SCT was assessed using the SIINFEKL/K^b^-specific antibody 25-D1.16 or anti-mouse β2m. *A*, a representative experiment showing 25-D1.16 staining of SCT transfected HeLa E1KO cells with (*red histogram*) or without (*blue histogram*) ERAP1 expression. *B*, the relative expression and percentage of 25-D1.16 stained WT or A-pocket variant SCT transfected HeLa E1KO cells in the presence or absence of ERAP1. *C*, cell surface expression of SCT assessed by anti-mouse β2m following transfection with ERAP1 (*red* = −ERAP1, *blue* = +ERAP1, filled histogram = negative control). The data are pooled from at least three experiments (*B*, error bars represent the S.D. of data; ***, *p* <0.001; **, *p* <0.01; *, *p* <0.05; ns, not significant).

In light of the similarities in overall structure and the likelihood that the selected mutations at positions 63, 66, or 167 result in weakened interactions between the peptide N terminus and MHC I, we proceeded to measure the impact of co-expressing ERAP1 with the variants on their cell surface expression and reactivity to 25-D1.16. Expression of WT (*002) ERAP1 (25) in SCT transfected HeLa E1KO resulted in a significant decrease in the levels of all A-pocket variants detected by 25-D1.16 (∼35–40%) (Fig. 4, *A* and *B*). By contrast, no difference in the levels of WT-SCT was observed when ERAP1 was expressed (Fig. 4, *A* and *B*). As expected, cell surface levels of all SCT variants in the presence of ERAP1, determined by anti-mouse β_2_M antibody, were similar, confirming that the reduction in 25-D1.16 staining was likely because of trimming of SL8 to IL7 and not because of decreased levels of SCT at the cell surface. This showed that mutation of the A-pocket increased the ability of ERAP1 to trim SCT-linked peptide, and indicated a link between A-pocket/peptide affinity and ERAP1 trimming of the N terminus.

### ERAP1 trimming of SCT reduces detection by B3Z T cells

**Figure 5.**
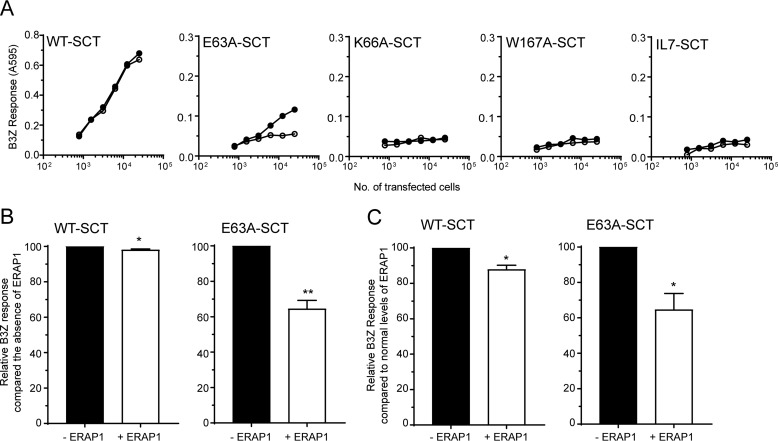
**ERAP1 reduces the stimulation of B3Z by single-chain trimer MHC molecules.** HeLa and HeLa E1KO cells were transfected with WT-SCT, A-pocket variants E63A-, K66A-, W167A-, or IINFEKL-SCT with or without overexpression of ERAP1. *A*, a representative experiment showing the stimulation of B3Z T cell hybridomas by the transfected SCT. *B*, the relative B3Z response of WT- and E63A-SCT in the presence or absence of ERAP1 in HeLa E1KO cells. *C*, the relative B3Z response of WT- and E63A-SCT in the presence or absence of additional ERAP1 in HeLa cells. The data are pooled from at least three experiments (*B*, error bars represent the S.D. of data; **, *p* <0.01; *, *p* <0.05).

To determine the effect of introducing the A-pocket mutations on antigen presentation to T cells we utilized B3Z T cell hybridomas. B3Z recognizes SL8 but not IL7 (Fig. S2) (37), and therefore illustrates how peptide overtrimming by ERAP1 can alter T cell responses to a dominant antigen. Unlike 25-D1.16, the B3Z T cell receptor was sensitive to mutations in the A-pocket, as all three variants were recognized less well compared with WT-SCT when transfected into HeLa E1KO, despite their equivalent expression at the cell surface (Figs. 4*A* and 5*A*). This disruption could be because of alteration of TcR contact regions not shared by the 25-D1.16 antibody (32, 38), either directly or indirectly, which is mediated via side chain rearrangement in K^b^ HC or SL8. In particular, the observed changes in the orientation of N(P_4_) side chains in the variants (Fig. 3) are consistent with previous observations that anti-SL8 TcRs are sensitive to alterations at P_4_, P_6_, and P_7_ (39). The least affected variant was E63A-SCT, so we examined the ability of this variant to stimulate B3Z when ERAP1 was expressed. In the presence of ERAP1, E63A-SCT showed a significant reduction in B3Z stimulation (∼35%), consistent with trimming of SL8 to IL7 (Fig. 5, *A* and *B*). By contrast, WT-SCT showed very little difference in B3Z stimulation when ERAP1 was present (Fig. 5, *A* and *B*). This difference was even detected against a background of endogenous ERAP1 expression in HeLa cells, where we observed a reduction in B3Z stimulation for both WT- and E63A-SCT after transfection of ERAP1, with the effect being most pronounced for the variant (∼35% reduction compared with ∼15% reduction) (Fig. 5*C*).

### Molecular dynamics simulations

The crystallographic and antigen presentation results indicate that although A-pocket mutations do not affect the overall structure of SCT, they still promote overtrimming of SL8 to IL7 by ERAP1. To investigate the possible mechanism underpinning this observation, we employed molecular dynamics (MD) simulations to examine the effect on dynamics of the WT-SCT and variant SCT at the nanosecond to microsecond timescale. The all-atom MD simulations were initiated from the coordinates of our four X-ray structures and were performed in explicit solvent for a total time of 0.51 μs for each system. To examine the effect of the linker on the dynamics of K^b^, we also carried out MD simulations after removing the PL linker between the SL8 and β_2_m. These calculations were performed using similar initial coordinates, simulation parameters, and total time for each system.

The peptide-binding domain of WT-SCT displayed remarkable stability as evidenced by the root mean square deviation (RMSD) of the corresponding C^α^ atoms with respect to the X-ray structure (Fig. S3*A*). The average value of 1.2 ± 0.1 Å suggests that the overall structure of the WT-SCT fluctuates around the crystallographic coordinates at the simulation timescale. By contrast, the SCT variants exhibited higher RMSD values of 1.7–1.8 ± 0.2–0.3 Å, a first indication of the effect of the mutated residues on the conformational dynamics of the antigen-binding groove. The peptide in both E63A- and K66A-SCT displayed low RMSD^Cα^ values (<1.0 Å), whereas SL8 in W167A-SCT deviated more from the initial position, with RMSD^Cα^ of ∼2.0 Å (Fig. S3*B*). In the untethered pMHC I (native and A-pocket mutants), the RMSD^Cα^ in the peptide-binding domain of K^b^ with respect to the crystallographic coordinates displayed similar values in the range of 1.3–1.4 Å (Fig. S3*C*). This was also the case for the SL8 peptide atoms in WT-, E63A-, and K66A-K^b^, which deviated by 2–3 Å (Fig. S3*D*). Remarkably, SL8 in W167A-K^b^ deviated significantly during the simulation and displayed RMSD^Cα^ of 6.8 ± 1.1 Å.

**Figure 6.**
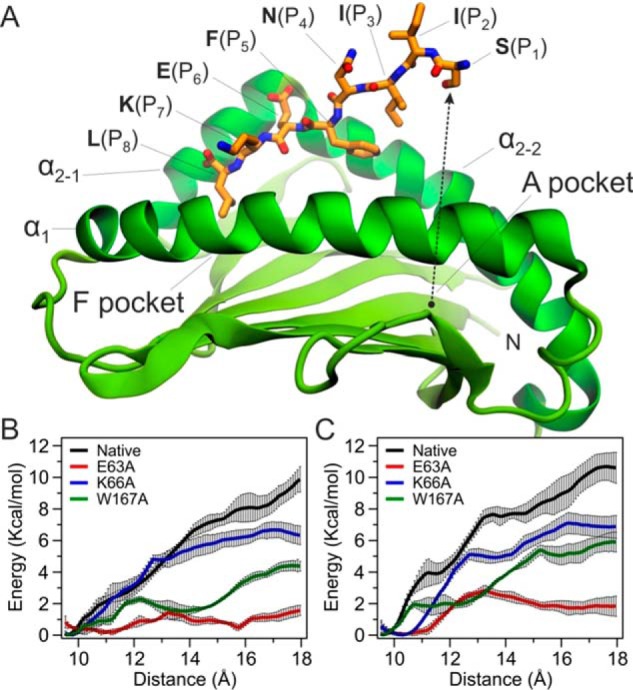
**MD simulations of single-chain trimers.** *A*, snapshot of the W167A-SCT peptide-binding domain from the unrestraint MD simulations exhibiting dissociation of the N-terminal half of SL8. The *dashed arrow* indicates the distance between S(P_1_) and the bottom of the A pocket (δ) that was employed as the reaction coordinate in free energy calculations. *B* and *C*, plots of the free energy change as a function of distance (δ) obtained from umbrella sampling calculations employing (*B*) the four SCTs and (*C*) the four corresponding untethered SL8/K^b^ complexes. *Error bars* indicate the S.D. at each point as extracted from four independent runs.

Examination of the MD trajectory of the untethered W167A pMHC I revealed that the N terminus of SL8 became detached from the A-pocket within 10 ns after equilibration of the system. Partial dissociation of the N-terminal portion of the peptide was observed, however complete dissociation did not occur at this timescale even when the anchor residue F(P_5_), initially buried inside the binding groove, turned toward the solvent-exposed face of the groove (Movie S1 and Fig. 6*A*), indicating the strong and preferential binding of the peptide C terminus. Although the rare event of partial peptide unbinding at the sub-microsecond timescale was only observed in the simulation of the W167A-SCT, this does not mean that similar dissociation events cannot occur in other pMHC I.

### Free energy calculations of the N terminus dissociation

Partial peptide dissociations have been observed previously in MD studies of HLA-B*27:05 (40), HLA-A*02:01 (41), as well as in MD simulations of the HLA-B*44:02 loaded with peptides truncated or modified at the C or N terminus (42). In addition, a “flapping” behavior of both the N- and C-terminal regions of the peptides has been reported in a large-scale MD study of HLA-B*08:01 in complex with 172 variants (single-point mutants) of the Epstein-Barr virus peptide FLRGRAYGL (43). These considerations prompted us to study the partial N terminus dissociation pathway using free energy calculations. To investigate the effect of A-pocket mutations on the peptide dynamics, we employed the umbrella sampling method and extracted the free energy profiles of N terminus partial dissociation for the WT-SCT and three variants. Umbrella sampling is performed through a series of restraint MD simulations that are carried out in small, overlapping steps (windows) along the pathway under study. In our case, the distance δ between the C^α^ atom of S(P_1_) and the center at the bottom of the A-pocket was selected as the collective variable (Fig. 6*A* and Fig. S4 and “Computational methods” for details). Simulations were initiated as close as possible to the crystallographic coordinates of the four SCTs and a harmonic potential was used to bias the reaction coordinate δ in 0.5-Å steps until the N terminus of S(P_1_) was completely exposed to the solvent. The free energy profiles (potentials of mean force) along the N terminus dissociation pathway were obtained from weighted histogram analysis of the collected data (Fig. 6*B*). All three SCT variants displayed lower free energy barriers of dissociation with respect to the WT-SCT and in the order of 3–8 kcal/mol. In particular, E63A-SCT exhibited the lowest free energy profile, followed by W167A-SCT, both of which displayed the lowest energy barriers at the midpoints of the dissociation path. To confirm these observations and exclude the potential bias introduced by the linker and the presence of the disulfide bond tethering the C terminus of the bound peptide, we calculated the corresponding potentials of the four SL8/K^b^ systems where the C(P_10_) in the linker is removed, untethering the peptide (Fig. 6*C*). These untethered SL8/K^b^ molecules exhibited comparable profiles to those observed for the tethered versions.

## Discussion

Using MHC I single-chain trimers we identified that alanine substitution of A-pocket MHC I residues contacting the N terminus of bound peptide resulted in ERAP1-dependent overtrimming of SL8 to IL7. Structures of these variants determined by X-ray crystallography showed that local side chain rearrangements of K^b^ and SL8 in the vicinity of the substituted residues in some cases retained favorable interactions between K^b^ and SL8 by including insertion of water molecules that preserved hydrogen bonds. Any reduction of peptide affinity because of weaker interaction between peptide N terminus and the A-pocket variants was not evident from the crystal structures. However, MD simulations and free energy calculations of SCT and untethered pMHC I clearly showed a strong correlation between the A-pocket variants and the ability of the peptide N terminus to partially dissociate and be accessible for ERAP1 trimming. Taken together, these data are consistent with the model proposed by Chen *et al.* (30), in which the partial dissociation of bound peptides from MHC I exposes their N terminus to trimming by ERAP1 while their C terminus remains deeply buried in the F-pocket.

MD simulations showed that partial dissociation of the peptide from K^b^ occurred at the N terminus with strong and preferential binding of the peptide C terminus. This finding is supported by the greater increase in *K*_off_ rates for N-terminal compared with C-terminal truncated peptides from D^b^ (44). In addition, a recent combined experimental and computational study of K^b^ demonstrated the prominent role of the C terminus in stabilization of the bound peptide and revealed that truncation of the peptide C terminus results in high flexibility of the F-pocket region, similar to that of the empty groove (45). These observations are consistent with the previously proposed model for trimming of N-terminally extended peptides where the C terminus is anchored into the F-pocket of the binding groove and the N terminus is available for trimming by ERAP1 (30).

In our experimental system, the increased stability afforded by tethering the peptide to MHC I has enabled observation of peptide trimming when bound to MHC I (17), but does not indicate whether it occurs during the normal processing of antigens. Chen *et al.* recently showed that both tethered and free peptide bound to HLA-B*08:01 were substrates for ERAP, indicating that trimming of MHC I-bound peptides could be a physiologically relevant mechanism (30). In addition, consistent with our observations, a recent MD study of peptide bound to HLA-B*44:02 showed that removal of the N-terminal amino acid caused the dissociation of the P_2_ amino acid, with further truncation leading to greater dissociation of the peptide from MHC I at the N terminus (42). The weaker interaction of the peptide N terminus predisposing to overtrimming and the greatly increased macroscopic dissociation rates of the resultant shorter peptides may therefore represent a quality control step similar to that for tapasin/PLC (46). This process would improve the half-life of pMHC I complexes at the cell surface through both generation of the correct peptide length and disposal of peptides that have poor N-terminal affinity.

The overtrimming of several epitopes by ERAP1 has been noted (11, 18–20), and it is likely that the steady-state generation of any given epitope is a balance between overtrimming and MHC I binding and egress of the pMHC I complex to the cell surface. The destruction of epitopes has been demonstrated for minor histocompatibility antigens where knockout of ERAAP increased the quantity of presented epitope (11), and for viral antigens (20). We have shown that ERAP1 overtrimming can have a significant impact on anti-tumor immunity in a preclinical model, where reduction of ERAAP protein in the murine CT26 tumor significantly increased generation of the immunodominant cryptic epitope (18). In addition, Keller *et al.* have shown that destruction of the immunodominant tumor antigen in melanoma, MART-1, by ERAP1 may affect the success of epitope-specific immunotherapy (19). These examples highlight the possibility of modulating of ERAP1 function therapeutically, for example with small molecules (18, 47) to enhance immunity. This is particularly pertinent in light of the fact that ERAP1 is functionally polymorphic in the human population (17), thus patients could be stratified according to their ERAP1 allotypes for their suitability to respond to targeted ERAP1 modulators. For example, patients expressing hyperactive ERAP1 might be likely to mount better responses to overtrimmed tumor-derived epitopes if given ERAP1 inhibitors, and conversely those expressing hypoactive allotypes if given ERAP1 activators.

Quite how ERAP1 might gain access to the N termini of partially dissociated MHC I–bound peptide substrates in such a way as to permit trimming to heptapeptide or shorter is unclear, and would require ERAP1 to adopt a conformation that is more open than observed in previously published crystal structures (26–28). Nevertheless, this study, when taken with other biological and structural data (29, 30, 42) indicate that ERAP1 can use the pMHC I complex as a substrate *in vitro* and *in vivo*, and that partial dissociation of MHC I–bound peptides can expose them to overtrimming. It will be important to learn how structural differences between naturally occurring ERAP1 allotypes relate to their enzyme activity, and whether their recognition of pMHC I as a substrate is a contributing factor.

## Experimental procedures

### Generation of SCT constructs

A SL8/K^b^ disulfide trap SCT has been described previously (17). To generate the A-pocket variants, site-directed mutagenesis of WT-SCT heavy chain amino acids at position Glu^63^, Lys^66^, and Trp^167^ to alanine residues was performed using the following primers (mutated nucleotides in italics): E63A, 5′-CGTATTGGGAGCGGG*CC*ACACAGAAAGCCAAGG and 3′-CCTTGGCTTTCTGTGT*GG*CCCGCTCCCAATACG; K66A, 5′-GGAGCGGGAGACACAG*GCC*GCCAAGGGCAATGAGC and 3′-GCTCATTGCCCTTGGC*GGC*CTGTGTCTCCCGCTCC; and W167A, 5′-GGCACGTGCGTGGAG*GC*GCTCCGCAGATACCTG and 3′-CAGGTATCTGCGGAGC*GC*CTCCACGCACGTGCC.

### Generation of ERAP1-deficient HeLa cells

ERAP1-deficient HeLa cells were generated using CRISPR/Cas9 according to Reeves *et al*. (25). ERAP1 knockdown was determined by immunoblot and successful clones were expanded and cultured.

### Cell lines, transfection, flow cytometry, and T cell activation

HeLa and HeLa E1KO cell lines were maintained in RPMI 1640 culture medium supplemented with 10% FCS, 2 mm l-glutamine, 1 mm sodium pyruvate, and 50 units/ml penicillin/streptomycin. B3Z T cell hybridoma (a kind gift from Prof. N. Shastri, University of California, Berkeley, CA) was cultured as described previously (8). HeLa and HeLa E1KO cells were transfected with 1 μg of each of the SCT constructs WT-SCT, IL7-SCT, E63A-SCT, K66A-SCT, and W167A-SCT, alongside either 1 μg ERAP1-pcDNA3.1 (described previously in Ref. 17) or empty vector (pcDNA3.1) control using FuGENE 6 (Roche). After 48 h, cell were harvested and assessed for SL8/K^b^ cell surface presentation by either flow cytometry or B3Z T cell activation. For cell surface expression of SCT, transfected HeLa or HeLa E1KO cells were stained for SL8/K^b^ (25-D1.16-FITC) and mouse anti-mouse β_2_m (S19.8, BD Biosciences, 555299) and analyzed by flow cytometry using BD FACS Canto II and FlowJo analysis software (BD Biosciences). For T cell activation, transfected cells were incubated overnight with the *LacZ*-inducible T cell hybridoma, B3Z, and assessed as described in Ref. 17.

### Immunoblotting

Expression of ERAP1 was determined by immunoblot. Both HeLa and HeLa Eko, transfected with and without ERAP1, were lysed in 0.5% Nonidet P-40, 150 mm NaCl, 5 mm EDTA, and 20 mm Tris, pH 7.4, supplemented with PMSF and iodoacetamide (Sigma-Aldrich). Proteins were separated by 10% SDS-PAGE and transferred to a nitrocellulose membrane (GE Healthcare). Immunoblots were probed with primary antibodies, goat anti-human ARTS1 (R&D Systems, AF2334) or mouse anti-GAPDH (Abcam, Ab8245) followed by HRP-conjugated secondary antibodies. Protein expression was determined using Supersignal West Pico or Femto Chemiluminescent Substrate (Thermo-Fisher).

### Expression of single-chain trimers

Disulfide-trap SIINFEKL/K^b^ single chain trimers (WT-SCT, a kind gift from Prof. Ted Hansen) were cloned and expressed in BL21 *Escherichia coli* using standard procedures. The expressed protein was purified from inclusion bodies, resolubilized in 8 m urea and refolded through rapid dilution at 4 °C (48). Concentrated refolded SCTs were purified using size-exclusion chromatography, His-tag purification, and anion exchange and stored in HEPES buffer.

### Determination of crystal structures

SCTs were crystallized with sitting drop vapor diffusion using a previously described screen for pMHC I crystallization (49). After cryoprotection with 30% v/v glycerol, crystals were frozen at 100 K and taken to beamlines IO4 and I24 at the Diamond Light Source, Oxford, UK, for collection of X-ray diffraction data. Data were processed with the automated Diamond xia2 software (50) and the CCP4 suite (51). Molecular replacement was performed with MOLREP using PDB coordinates 2QRI (33) for the WT-SCT, E63A-SCT, and K66A-SCT structures, and the solved structure of E63A-SCT for W167A-SCT molecular replacement. Refinement was carried out in PHENIX (52) and subsequent rounds of model building using COOT (53). Optimal hydrogen bonding was calculated using the WHAT_IF server (54). The structures were deposited on PDB with accession codes 5OQF (WT-SCT), 5OQI (E63A-SCT), 5OQH (K66A-SCT), and 5OQG (W167A-SCT).

### Computational methods

The four disulfide-trapped single-chain MHC I simulation systems were based on the crystallographic structures described herein. Only protein atoms were retained and the unresolved residues of the linker between the peptide and the β_2_m (Gly^14^–Ser^23^) were modeled on the basis of the SL8-SCT (Y84C) structure determined previously (PDB ID: 2QRT) (33). The second linker between the C terminus of β_2_m and the N terminus of the heavy chain was not modeled as it is not resolved in any X-ray structure to data and is not expected to affect the peptide dynamics significantly. Four untethered SL8/K^b^ complexes were prepared by removing all atoms of residues Gly^9^–Ser^13^ of the linker that were resolved from the corresponding crystal structure of the SCT, and then the engineered Y84C was changed back to tyrosine.

Hydrogen atoms were added after calculating the protonation state of the ionizable groups at physiological pH (7.4) using the h++ v3.1 server with default parameters at pH 7.4 (55). In particular, histidine residues 3 and 93 of the heavy chain were set as positively charged, His^68^ of β_2_m was protonated at N^δ1^, whereas all other histidine residues were protonated at N^ϵ2^. The proteins were solvated into truncated octahedral boxes with TIP3P waters extending up to 12 Å around the solute; the appropriate number of Na^+^ and Cl^−^ ions was added so as to neutralize the total charge of each system and simulate an ionic strength of 0.15 m, and the AMBER *ff14SB* force field parameters (56) were applied.

Molecular dynamics simulations were performed with the GPU-accelerated version of PMEMD (57) at the Iridis4 supercomputer facility (University of Southampton). The MD parameters and equilibration protocol employed were the same as described in Ref. 58. Production runs were performed for 500 ns for each system in the NPT ensemble (*T* = 300 K; *p* = 1 bar). Trajectory snapshots were collected every 2 ps for further processing excluding the initial 10-ns equilibration period. Visual inspection of the trajectories and rendering of figures and movies was performed with VMD v1.9.3 (59).

Umbrella sampling calculations were performed for the four SCTs and the four untethered SL8/K^b^ systems. The potential of mean force (PMF) of the peptide N terminus dissociation was calculated using harmonic distance restraints between the C^α^ atom of the N-terminal S(P_1_) and the center of mass of the C^α^ atoms of residues 5, 6, 7, 26, 27, 28, 99, 100, and 101 at the base of the A-pocket (Fig. S4). The reaction coordinate (δ) was divided in 16 windows of 0.5-Å steps, starting from δ = 10 Å until complete dissociation of the N-terminal residue at δ = 17.5 Å. Calculations were initiated from the solvent equilibrated systems at 1 ns and before releasing the positional restraints on the C^α^ atoms, so all systems were as close as possible to the experimental structures. Equilibration was carried out for 5 ns in each window and the structure obtained at the last frame was used as the starting structure of the subsequent window. Four independent MD simulations of 5 ns were performed in each window with force constants adjusted empirically so as to reach convergence of the resulting PMFs within at least 1 kcal/mol and obtain a satisfactory overlap of the reaction coordinate distributions between adjacent windows (Fig. S5). In cases where convergence was not satisfactory because of large conformational space that was not sampled, simulations were extended to 10 ns. The unbiased probability distributions of the distances and the PMF were obtained using the weighted histogram analysis method (60) with convergence tolerance of 0.001 Å. The errors at each point in the PMF plots were calculated as the S.D. from the four independent production sets.

### Statistical analysis

Two-way paired Student's *t* test was performed for analysis of differences between experimental groups (GraphPad Prism).

## Author contributions

A. P., E. R., M. B., H. M., L. D., and G. C. data curation; A. P., E. R., M. B., H. M., and J. M. W. formal analysis; A. P., E. R., and M. B. investigation; A. P., E. R., and L. D. methodology; A. P., E. R., M. B., H. M., L. D., G. C., T. E., J. M. W., and E. J. writing-review and editing; E. R. and E. J. validation; T. E., J. M. W., and E. J. conceptualization; T. E., J. M. W., and E. J. supervision; T. E., J. M. W., and E. J. funding acquisition; T. E., J. M. W., and E. J. project administration; E. J. writing-original draft.

## Supplementary Material

Supporting Information

## Footnotes

3 The abbreviations used are:

MHC I: major histocompatibility complex class I
pMHC I: peptide-bound MHC I
ER: endoplasmic reticulum
ERAP1: ER aminopeptidase 1
TAP: transporter associated with antigen processing
Tpn: tapasin
PLC: peptide-loading complex
CTL: cytotoxic T lymphocyte
HC: heavy chain
β_2_m: beta-2-microglobulin
SCT: single-chain trimer
SL8: SIINFEKL
IL7: IINFEKL
MD: molecular dynamics
PMF: potential of mean force
MD: molecular dynamics
RMSD: root mean square deviation
Am sulfate: ammonium sulfate
bis-tris: 2-[bis(2-hydroxyethyl)amino]-2-(hydroxymethyl)propane-1,3-diol.
