# Supplementary material for: The partial dissociation of MHC class I–bound peptides exposes their N terminus to trimming by endoplasmic reticulum aminopeptidase 1
Source: J Biol Chem. 2018 Mar 29;293(20):7538–48. doi: 10.1074/jbc.RA117.000313 (PMC5961055; doi:10.1074/jbc.RA117.000313)
Supplement: Supporting Information [file supp_293_20_7538__index.html]

The partial dissociation of MHC class I bound peptides exposes their N terminus to trimming by endoplasmic reticulum aminopeptidase 1 — ERAP1 trimming of MHC I-bound peptides — The partial dissociation of MHC class I–bound peptides exposes their N terminus to trimming by endoplasmic reticulum aminopeptidase 1 — ERAP1 trimming of MHC I–bound peptides — Supporting Information 

# The partial dissociation of MHC class I–bound peptides exposes their N terminus to trimming by endoplasmic reticulum aminopeptidase 1

## Supporting Information

- Supplemental data - Supplemental movie and figures to support the manuscript
