# Supplementary material for: The partial dissociation of MHC class I–bound peptides exposes their N terminus to trimming by endoplasmic reticulum aminopeptidase 1
Source: J Biol Chem. 2018 Mar 29;293(20):7538–48. doi: 10.1074/jbc.RA117.000313 (PMC5961055; doi:10.1074/jbc.RA117.000313)
Supplement: Supporting Information [file supp_RA117.000313_132957_2_supp_101902_p6032r.pdf]

The amino-terminus binding pocket of MHC class I impacts the ability of ERAP1 to trim bound peptides

Athanasios Papakyriakou[1,2,*], Emma Reeves[2,3,*], Mary Beton[1,2], Halina Mikolajek[1,2], Leon Douglas[2], Grace Cooper[2,3], Tim Elliott[2,3], Jörn M. Werner[1,2], Edward James[2,3]

From the [1]Centre for Biological Sciences, Faculty of Natural & Environmental Sciences, University of Southampton, UK; [2]Institute for Life Sciences, University of Southampton, Southampton, UK; [3]Centre for Cancer Immunology, Faculty of Medicine, University of Southampton, UK

Supplemental Data

Supplemental Movie 1. Animation from a 0.5-μs unrestraint MD simulation of SL8/K^b W167A mutant showing the partial, N-terminus peptide dissociation. The peptide binding domain is only shown helices as a blue surface, the β-sheet base as green surface, while the red surface indicates the mutated position. The peptide is shown with orange C, blue N and red O sticks.

(For the submission please download the movie at: https://www.dropbox.com/s/prvnnoiw4l8ix18/H-2Kb_W167A_MDs.mpg?dl=0)

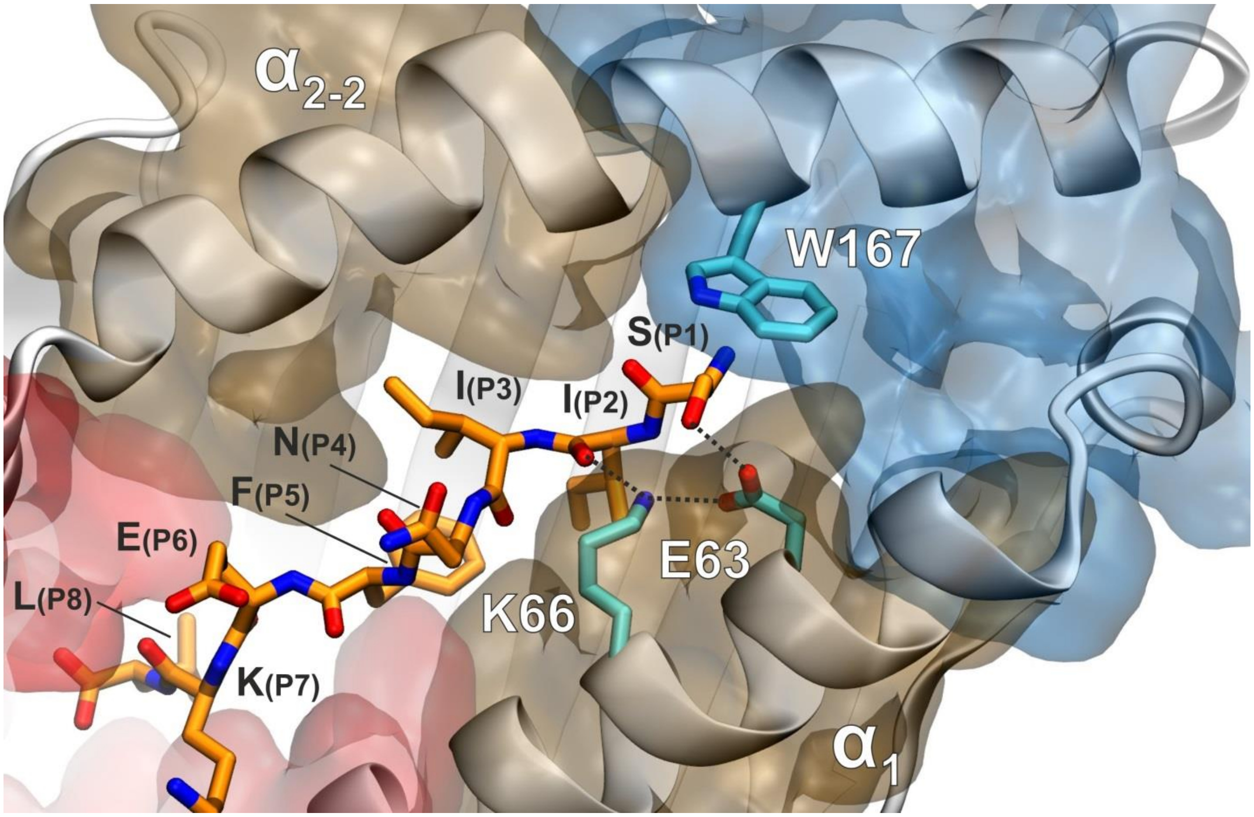

Supplemental Fig. S1. Close-up view of the A and C pocket regions (blue and brown surfaces, respectively) in the X-ray structure of SL8-SCT, illustrating the hydrogen bonding interactions between the S(P_1)–E63 and I(P_2)–K66. The indole group of W167 caps off the N-terminal amino group of the peptide, which is also stabilized via two hydrogen bonds with the phenol groups of Y7 and Y171 (shown in Fig. 3).

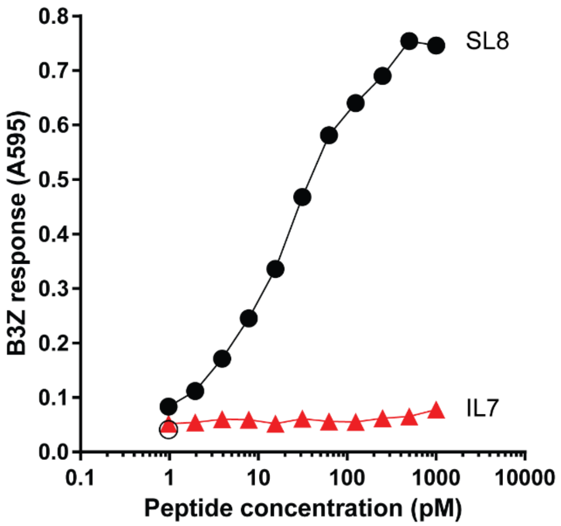

Supplemental Fig. S2. Peptide stimulation of B3Z. H2-K^b expressing K89 cells were incubated with titrating concentrations of SL8 (black circles), IL7 (red triangles) peptides or no peptide (open circle) and B3Z T cell hybridoma added. After overnight incubation, stimulation of B3Z was assessed using CPRG and measured at 595nm wavelength.

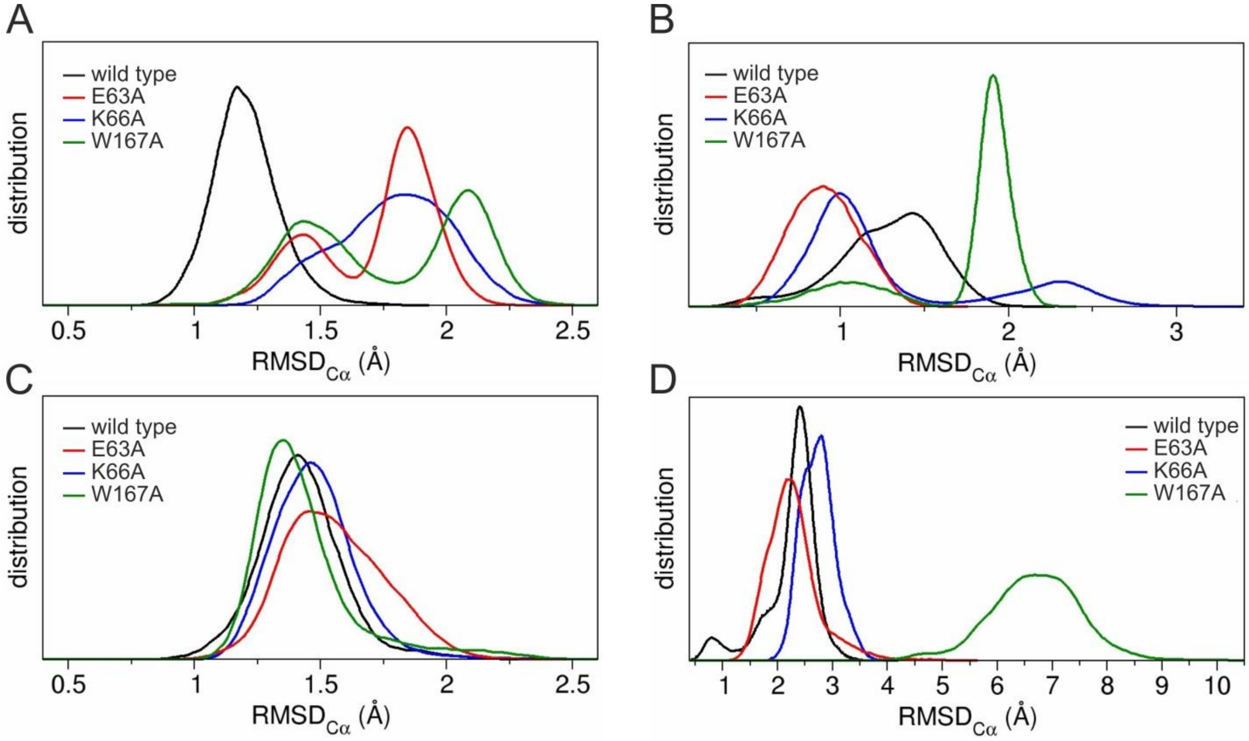

Supplemental Fig. S3. Distributions of root-mean-square deviation of C^{α} atoms ($RMSD_{C\alpha}$) from the initial, crystallographic structures, obtained from 0.5 μs unrestraint all-atom MD simulations: (A) the peptide binding domain residues 1–180, and (B) the SL8-only C^{α} atoms of the four SL8-SCTs. (C) and (D) are the same as (A) and (B) for the corresponding untethered SL8/K^{b} systems.

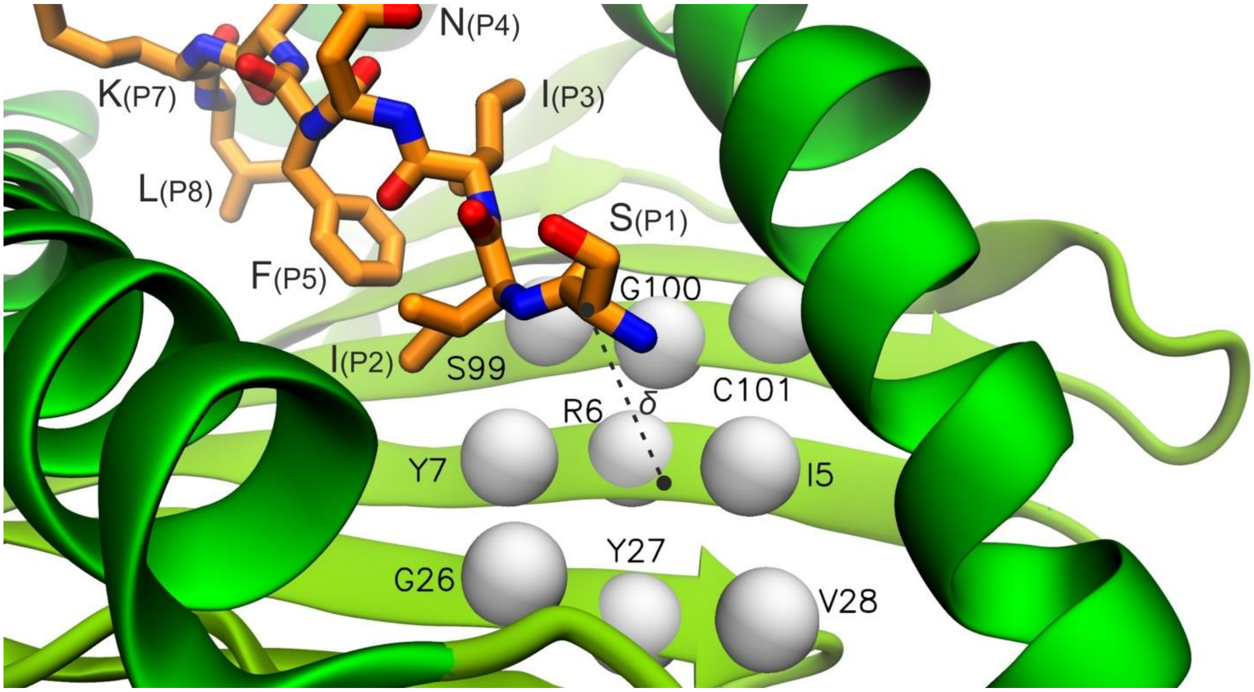

Supplemental Fig. S4. Definition of the collective variable employed in the umbrella sampling calculations as the distance (δ) between the C^{α} atom of the N-terminal residue of the peptide and the center of mass of the C^{α} atoms in the base of the A pocket indicated with white spheres and labelled.

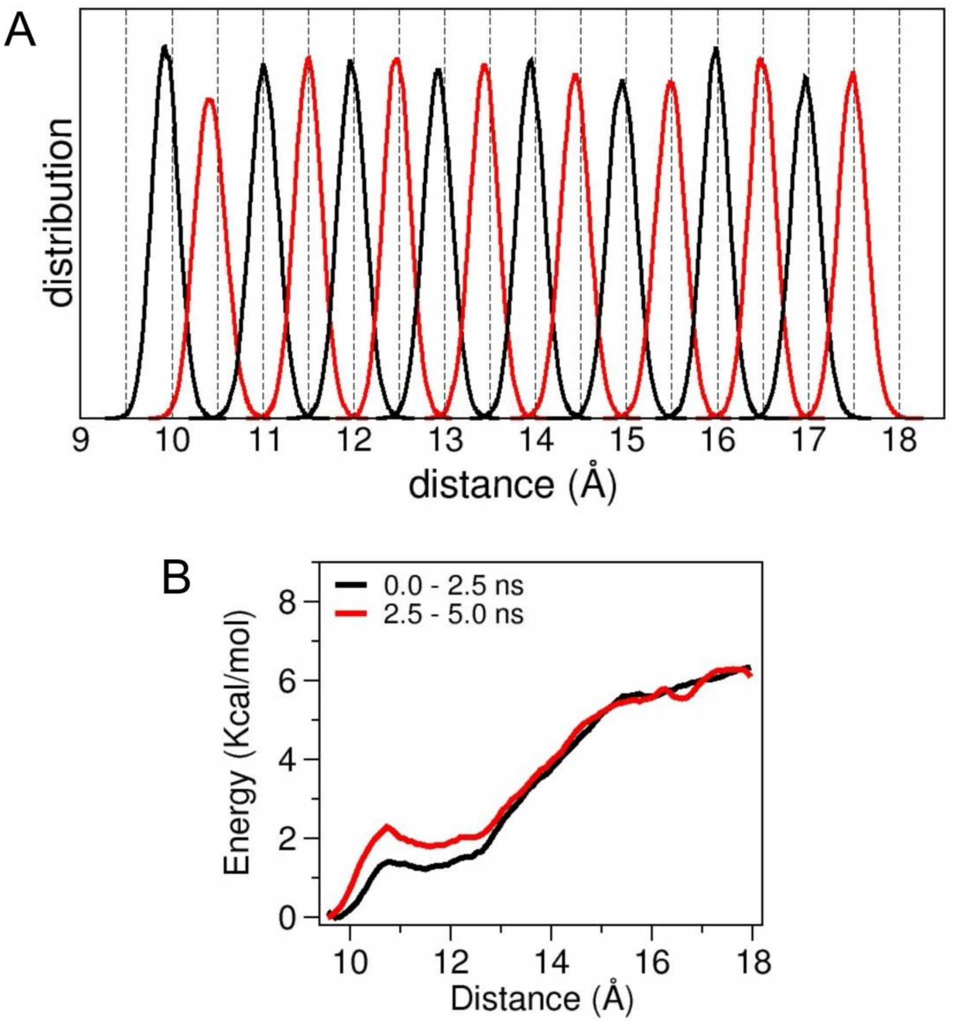

Supplemental Fig. S5. Characteristic biased distributions of the collective variable (distance, δ) exhibiting the overlap between successive windows (A) and the corresponding free energy profiles (PMF) displaying the convergence achieved by processing the 5-ns MD data in two halves (B). These results were taken from one out of the four independent simulations of the untethered SL8/K^b W167A mutant.
